# What happened to the concept of adolescence crisis?

**DOI:** 10.1007/s00787-020-01660-y

**Published:** 2020-10-10

**Authors:** Robert Waltereit, Anne Uhlmann, Stefan Ehrlich, Veit Roessner

**Affiliations:** 1 Department of Child and Adolescent Psychiatry, University Medical Center Göttingen, Von-Siebold-Str. 5, 37075 Göttingen, Germany; 2 Department of Child and Adolescent Psychiatry, Faculty of Medicine, University Hospital Carl Gustav CarusTU Dresden, Fetscherstrasse 74, 01307 Dresden, Germany; 3 Division of Psychological and Social Medicine and Developmental Neurosciences, Faculty of Medicine, TU Dresden, Fetscherstrasse 74, 01307 Dresden, Germany

Adolescence is a critical period in human development and a time when many psychiatric disorders emerge [1]. In the older literature, the term “adolescence crisis” appeared frequently; today it is still in some clinicians’ vocabulary. It appears at first glance as a suitable semantic combination describing the time period in life as well as its subliminal and transient psychiatric burden. However, the previously commonly used term is now seen with scepticism [2]. What was the idea behind the concept of adolescence crisis, why was it left behind and what could be a useful legacy?

The term “adolescence crisis” appears to have originated from the European child and adolescent psychiatric tradition. It was defined as a “pragmatic term for very heterogeneous psychopathological patterns characterized by joint onset and in their course usually tempestuous and cluttered with symptoms” whilst at the same time, representing “normal variants of mental experience and behaviour during adolescence” [3]. Thus, in an “adolescence crisis” the spectrum of symptoms can be met partly or even fully. The respective symptoms are not interpreted as an emerging psychiatric disorder, as the individual is transiting adolescence with the symptoms accordingly being perceived as transient. In our opinion, this concept of “adolescence crisis” attempts to address two fundamental issues in developmental psychiatry. First, which nosological entity is characterized by (1) strong fluctuation of symptoms, (2) short-term changes or even remission without treatment, and (3) an only partial match with symptoms of major psychiatric disorders in the framework of established classification systems with their focus on adult psychiatry? Second, which biological, psychological or social mechanisms underlie the pattern of current symptoms? These challenges raise further questions. For example: Is the underlying mechanism specific for an emerging major psychiatric disorder, hence requiring early diagnosis and treatment?, Are the symptoms a sub-clinical manifestation or a ‘forme fruste’ of a major psychiatric disorder but with spontaneous remission over time?, and Or do we see a pattern of ultimately normal or non-pathological symptoms within the spectrum of physiological developments during puberty but in an uncommon combination?

Here, the concept of “adolescence crisis” builds a bridge to the “identity crisis”, a psychoanalytic term shaped by Erik Erikson. Identity crisis is part of stage 5 in Erikson’s “Stages of the Life Cycle”, ranging from eleven years until the end of adolescence. Stage 5 is characterized by “the main task to develop a sense of identity”. Later it has been postulated that “an identity crisis occurs at the end of adolescence. (…) Role confusion may manifest in such behavioural abnormalities as running away, criminality, and overt psychosis. Problems in gender identity and sexual role may become manifest at this time” [4]. Psychoanalysis is now less prominent and this may have influenced the decreasing use of the term “adolescence crisis”. Moreover, increasing streamlining of classification schemes prevents the use of non-standardized diagnostic terms—irrespective of their usefulness or limitations.

Additional explanations for the present reluctance in using the term “adolescence crisis” may arise from several developments over the last decades regarding psychotic and personality disorders. During adolescence, emerging psychotic and personality disorders, including schizophrenia, bipolar disorder and borderline personality disorder, are major differential diagnoses next to mood and anxiety disorders [5–12]. Recent and very important research has sharpened the understanding of these conditions [13, 14], which differentiates them from normal development including puberty. Many of these conditions have their onset in adolescence rather than in adulthood, including subclinical precursors as a prodromal state. This high prevalence of early onsets could put increased pressure on the clinician to make an early diagnosis of a major psychiatric disorder instead of practicing watchful waiting as the consequence of alternatively ‘diagnosing’ an “adolescence crisis”. Moreover, early diagnosis is now followed by early treatment, as there has been remarkable progress in the therapeutic principles of several of these early onset conditions.

In psychotic disorders, at-risk-states can now be better detected, thus allowing diagnoses at the earliest time point possible. We now know that the (short) duration of untreated psychosis (DUP) is pivotal for maintenance of a psychosocial functional level and quality of life in patients with schizophrenia [15]. But on closer inspection, most mentioned studies have been performed with a focus on emerging schizophrenia and related psychotic disorders and not on “adolescence crisis” or both. Therefore, their still unexplained variance as well as limited sensitivity and specificity on the long run together with increasing knowledge about adverse consequences of longer-term medication even of the better tolerated, newer antipsychotic agents [16] should encourage researchers to compare both concepts in studies regarding them as equal alternative hypotheses [17].

Compared to psychotic disorders, a possibly even more revolutionary change has happened for the concept of personality disorders. In the older literature, personality disorders were seen as static, life-long conditions with poor responses to therapy. Thus, there was a great reluctance to diagnose personality disorders in adolescents, to avoid stigmatization as well as to avoid the individual label of unlikely therapeutic benefit. In the last years, it has been understood that first symptoms of personality disorder emerge early in life, showing greater fluctuations over time in both adolescence and early adulthood. Moreover, it could be proven that they can be treated with good efficacy, especially borderline personality disorder with dialectical behaviour therapy (DBT) [18].

Taken together, it remains a constant challenge to identify and classify the symptoms of emerging psychiatric disorders within the colourful picture of typical adolescence. Nevertheless, the psychiatric view still appears to be driven primarily by diagnostic entities — is there psychosis, personality disorder, obsessive–compulsive disorder etc. in adolescence? However, in adolescence psychotic and personality disorders represent only a fraction of possible psychiatric disorders and other, “transdiagnostic” behaviours such as disruptive, self-harm and suicidal behaviours [16, 19, 20] still await more attention.

Hence, we would like to propose a more differentiated view on adolescence psychiatry following our editorial from 2015 “Neurobiological research in child and adolescent psychiatry: does the pendulum swing back to more attention on developmental psychopathology?” [21]. The idea of an “adolescence crisis” wants to stress the notion that in adolescents, there are manifold impairments in psychosocial functioning, but these are not necessarily associated with an emerging psychiatric disorder, or at least, we do not know yet. Could the idea of an “adolescence crisis” generate new perspectives on psychiatric research in adolescence, if we did not primarily look for psychiatric disorders, but for psychosocial functioning instead? This would mean to identify those adolescents who have a declining school performance, have trouble in peer groups, experience severe family conflicts and who lag behind in psychosocial terms including psychosexual development. Why do these individuals lag behind in developmental steps? What psychopathology is associated? What is the longitudinal outcome of these adolescents? Studying such populations of adolescents without predefined focus on emerging psychiatric disorders might generate novel research views and directions — and might be a useful legacy of the “adolescence crisis” concept.
